# Resection of Noncontrast-Enhancing Regions Deteriorated the Immunotherapeutic Efficacy of HSPPC-96 Vaccination in Treating Glioblastoma

**DOI:** 10.3389/fonc.2022.877190

**Published:** 2022-05-18

**Authors:** Xiaohan Chi, Yi Wang, Chunzhao Li, Xijian Huang, Hua Gao, Yang Zhang, Nan Ji

**Affiliations:** ^1^ Department of Neurosurgery, Beijing Tiantan Hospital, Capital Medical University, Beijing, China; ^2^ China National Clinical Research Center for Neurological Diseases, Beijing, China; ^3^ Cure & Sure Biotech Co., LTD, Shenzhen, China

**Keywords:** glioblastoma, extent of resection, immunotherapeutic efficacy, cancer treatment vaccine, HSPPC-96

## Abstract

Surgical resection remains a first-line therapy for glioblastoma multiforme (GBM). Increased extent of resection (EOR) of noncontrast-enhancing regions in T2-weighted MRI images (T2-EOR) provides a survival benefit for GBM patients receiving standard radio/chemotherapy. However, whether it also improves immunotherapeutic outcomes remains unclear. We calculated the T2-EOR by comparing the preoperative and postoperative MRI T2 hyperintensity outside the enhancing tumour and correlated the T2-EOR with immunological and clinical outcomes from our published early-phase trial of heat shock protein peptide complex-96 (HSPPC-96) vaccination in treating a cohort of 19 patients with newly diagnosed GBMs (NCT02122822). Patients with higher T2-EOR exhibited shorter progression-free survival (PFS) (HR 11.29, p=0.002) and overall survival (OS) (HR 6.5, p=0.003) times than patients with lower T2-EOR. T2-EOR was negatively correlated with the levels of tumour specific immune response (TSIR) post-vaccination (R=-0.725, p<0.001) and absolute TSIR increase from pre- to post-vaccination (R=-0.679, p=0.001). Multivariate Cox regression models revealed that higher T2-EOR represented an independent risk factor for PFS (HR 19.85, p=0.0068) and OS (HR 21.24, p=0.0185) in this patient cohort. Taken together, increased T2-EOR deteriorated immunotherapeutic outcomes by suppressing TSIR, suggesting the potential of T2-EOR as an early biomarker for predicting the immunotherapeutic efficacy of HSPPC-96 vaccination.

## Introduction

Glioblastoma multiforme (GBM), the most common and deadliest primary malignant brain tumour in adults, presents a great challenge for neurooncologists and neurosurgeons, as the outcomes of GBM patients have not greatly improved over the past two decades (1). The median survival for GBM is still limited to 15-20 months after standard-of-care treatment, which includes surgical resection, radiotherapy, temozolomide-based chemotherapy (2) and the recently developed tumour treating fields therapy (3). Among these therapies, surgical resection remains a primary treatment option for GBM. The extent of resection (EOR) greatly impacts clinical outcomes, with gross total resection of contrast-enhancing regions in GBMs significantly extending progression-free survival (PFS) and overall survival (OS) times (4, 5) compared with subtotal resection. Meanwhile, the rapid progress in novel intraoperative assistive technologies (6–8) has facilitated the transition from gross total resection to supratotal resection (SpTR), which expanded the EOR into noncontrast-enhancing regions outside the contrast-enhancing regions (9, 10). The noncontrast-enhancing region usually contains a small number of tumour cells, rendering it a region susceptible to tumour recurrence (11, 12). Therefore, recent clinical findings have supported the view that SpTR improves PFS and OS in a fraction of GBM patients receiving standard-of-care radiotherapy and chemotherapy (9, 13).

The last decade has witnessed unprecedented progress in cancer immunotherapies, which have already been approved as primary treatment options for a variety of malignancies (14–16). In glioma, immunotherapies, including immune checkpoint blockade, dendritic cell vaccines, peptide vaccines as well as chimeric antigens receptor-T cell therapy, have also generated encouraging early-clinical results in a fraction of patients, thus bringing hope for GBM patients (17–22). Heat shock protein peptide complex-96 (HSPPC-96) is a tumor-derived protein complex that comprises heat shock protein glycoprotein 96 kDa (gp96) and its binding tumor antigen derived peptides (23, 24). HSPPC-96 can be taken up directly by antigen-presenting cells (dendritic cells) and then trigger specific antitumor responses (25, 26), rendering it available as a cancer-treatment vaccine (27–29). Several studies have demonstrated its preliminary effectiveness in treating recurrent (17) and newly diagnosed GBMs (19). We previously conducted a single-arm, phase I clinical study that enrolled a total of 20 patients to evaluate its safety and efficacy in treating newly-diagnosed GBMs (NCT02122822) (19). We observed a median PFS of 11.0 months and a median OS of 31.4 months in the 19 patients with complete follow-up information. Meanwhile, we uncovered that the level of tumour-specific immune response (TSIR) post-vaccination were closely associated with the survival time (19). The median OS time for patients with high TSIR post-vaccination was >40.5 months as compared with 14.6 months for patients with low TSIR post-vaccination (Hazard ratio, 0.25; 95% CI, 0.071–0.90; P = 0.034) (19). We further revealed the presence of specific intratumoral T cell receptor clones were capable of predicting a durable response to the immunotherapy (30). However, whether SpTR improves outcomes in immunotherapies such as HSPPC-96 vaccination is still unclear.

In this study, we reviewed the clinical and immunological data, as well as the preoperative and postoperative (48-72 hours after surgery) MRI images, of patients in the HSPPC-96 vaccinated cohort (19). We calculated the EOR of noncontrast-enhancing regions in T2-weighted MRI images (T2-EOR) by comparing preoperative and postoperative T2-weighted MRI images and then correlated the T2-EOR with the TSIR levels as well as clinical outcomes to explore the impact of SpTR on the effectiveness of GBM immunotherapy.

## Materials and Methods

### Patients

We previously conducted a single-arm phase I clinical trial to determine the safety and efficacy of HSPPC-96 vaccination, combined with standard adjuvant radiotherapy and chemotherapy, in treating newly diagnosed GBM patients (19). A total of 20 patients with histologically confirmed GBMs were enrolled and received a total of six doses of the vaccine. The start of vaccination was concurrent with the beginning of adjuvant temozolomide chemotherapy. Vaccines were administered *via* subcutaneous injection in 25-μg doses every week for 6 weeks. Cyclophosphamide (400 mg) was given through intravenous injection before each HSPPC-96 vaccination (19). The primary endpoint of this study was PFS at 6 months and the frequency of adverse events. The secondary endpoints included OS and PFS at the end of the study and TSIR during the vaccinations. The clinical trial was approved by the ethics committee of Beijing Tiantan Hospital (JS2012-001-03) and was registered at ClinicalTrials.gov (NCT02122822) and http://www.chictr.org.cn/enindex.aspx (ChiCTR-ONC-13003309). Written informed consent was obtained from all participants. All the included patients underwent gross total resection of contrast-enhancing regions in the tumour and had a Karnofsky performance status (KPS) scale (range 0-100%) score of ≥ 70% before vaccination (19). Finally, 19 patients with available follow-up information for clinical efficacy analysis were included in the current study.

### Enzyme-Linked Immunospot Assay

TSIR was evaluated by the number of stimulated peripheral blood mononuclear cells (PBMCs) in response to autologous tumour lysate in an IFN-γ release ELISPOT assay, as previously reported (19). In the assay, PMA (Millipore Sigma) was included as a positive control, and wells without added tumour lysates or PMA were used as negative controls. IL-2 (Millipore Sigma) was also added to each well (50 IU/ml). A total of 3 × 10^5^ PBMCs were added to each well. Spots were counted using an ELISPOT reader (Cellular Technology Ltd.). The number of PBMCs responding to tumour lysate, as measured by IFN-γ release, was calculated by subtracting the background number of IFN-γ spots in the negative control well.

### Imaging Studies

Preoperative MRI images were obtained within 14 days before surgery, and postoperative MRI images were obtained within 3 days after surgery. The noncontrast-enhancing regions were represented as T2-hyperintense areas outside the contrast-enhancing lesions in T1-weighted images, termed T2-nonenhancing regions. After the T2-nonenhancing regions were manually outlined, the volume was automatically calculated with Neusoft PACS (version 5.5, Neusoft, China) software, which has been shown to be reproducible and accurate for tumour volume estimation in previous studies (9, 31).

### Statistical Analysis

Categorical data were compared using Fisher’s exact test. Continuous data were compared using analysis of variance (ANOVA) or Wilcoxon nonparametric tests. Pearson correlation tests were applied to determine the association between T2-EOR and TSIR indicators. PFS and OS were estimated by the Kaplan–Meier method, and the difference between groups was assessed by the log-rank test. Multivariate Cox regression models were fitted to identify independent risk factors for survival. Statistical significance was set as a 2-tailed p value of less than 0.05. All statistics were analysed using R (version 4.1.2) statistical software.

## Result

### Baseline Characteristics and T2-EOR Evaluation

**Figure 1:**
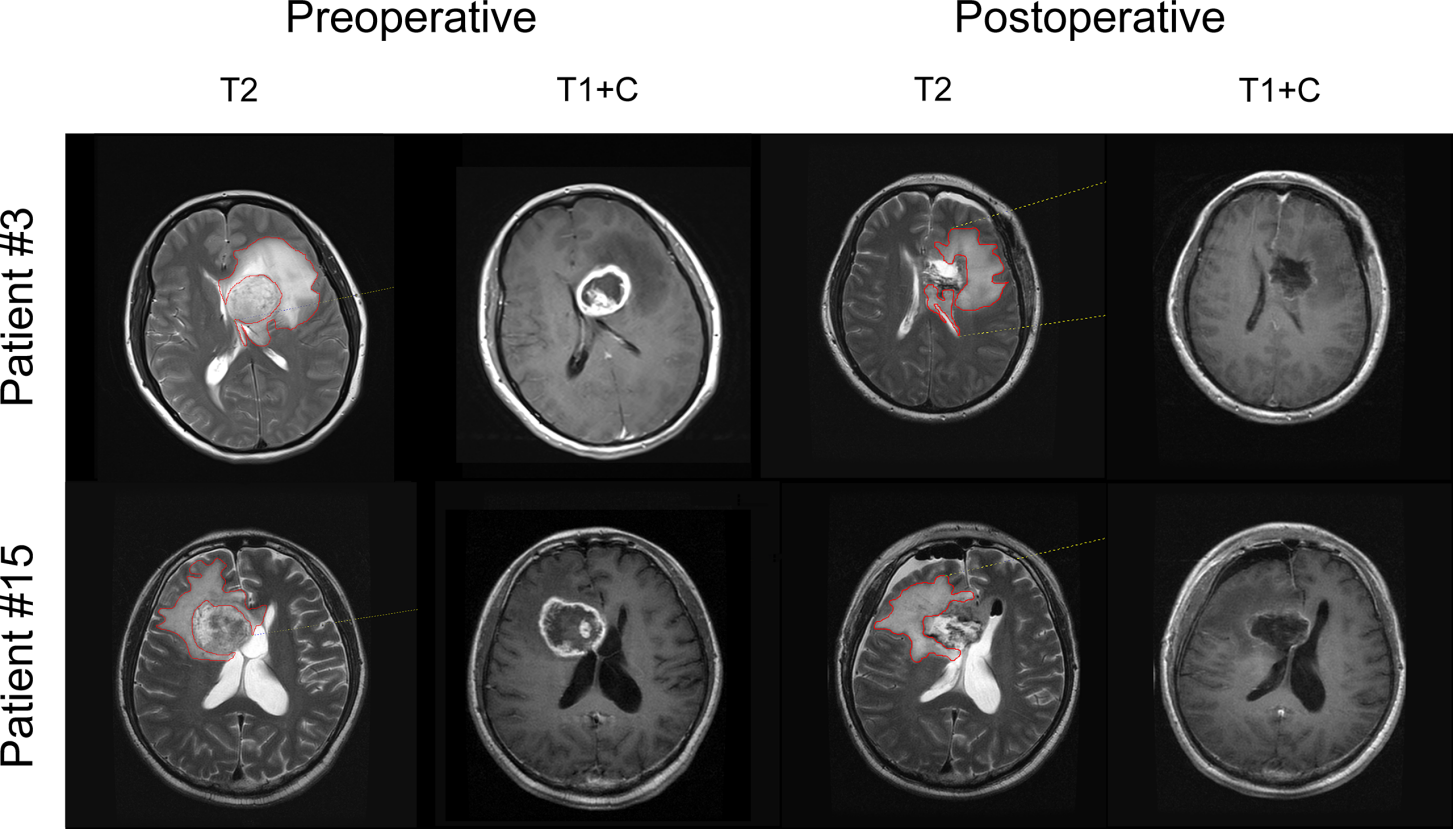
T2-EOR determination on preoperative and postoperative MRI images. The noncontrast-enhancing regions were represented as T2-weighted hyperintense areas outside the T1-weighted contrast-enhancing lesions and were termed T2-nonenhancing regions. T2-nonenhancing regions were manually outlined in red at each level, and the volume was automatically calculated by Neusoft PACS software. T2-EOR was defined as the preoperative-to-postoperative change in T2-nonenhancing region volume divided by the preoperative T2-nonenhancing region volume.

Recent clinical studies have shown the benefits of SpTR of noncontrast-enhancing regions, not just gross total resection of enhancing lesions, for survival outcomes in GBM patients (9, 10, 13). The noncontrast-enhancing regions were represented as T2-hyperintense areas outside the contrast-enhancing lesions and were termed T2-nonenhancing regions (**Figure 1**). Since the survival benefit of SpTR was achieved in the context of patients receiving standard-of-care radiotherapy and chemotherapy after surgery, we then wondered whether SpTR could improve the immunotherapeutic outcomes. To answer this question, we reviewed the clinical and immunological data, as well as the preoperative and postoperative (48-72 hours after surgery) MRI images, of an early-phase trial that evaluated the safety and preliminary efficacy of HSPPC-96 vaccination in a cohort of 19 patients with newly diagnosed GBMs (19). The baseline epidemiological and clinical characteristics, including sex, age at diagnosis, KPS scale score (range 0-100%), MGMT promoter methylation status, IDH 1/2 mutations and TERT promoter mutations, were reported in our previous study (**Supplementary Table 1**) (19).

Gross total resection of contrast-enhancing tumour areas was achieved in all patients (**Supplementary Table 1**). However, the median preoperative and postoperative volumes of T2-nonenhancing region were 75.69 cm^3^ (range 40.57-153.25 cm^3^) and 45.11 cm^3^ (range 24.60-154.15 cm^3^), respectively, resulting in a 40.1% median T2-EOR (range -12.7%-67.8%), as shown in **Supplementary Table 1**. Based on the T2-EOR, we divided the 19 included patients into high (T2-EOR ≥ median) and low (T2-EOR < median) T2-EOR groups. We compared all the baseline features between the two groups but did not observe any significant differences (**Supplementary Table 2**).

### Patients With Increased T2-EOR Exhibited Poorer Prognosis

**Figure 2:**
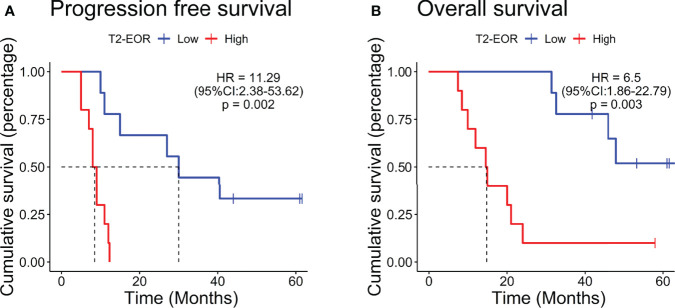
Patients with increased T2-EOR exhibited poorer prognosis. Kaplan–Meier estimates of **(A)** progression-free survival and **(B)** overall survival in 19 GBM patients, which were divided into the high (T2-EOR ≥ median) and low (T2-EOR < median) T2-EOR groups. The log-rank test was applied to estimate the difference. Vertical lines indicate time points at which patients were censored.

The median PFS was 11.0 months, and the median OS was 31.4 months in this vaccinated cohort (30). Surprisingly, in contrast to the standard-of-care treatment, increased T2-EOR deteriorated the immunotherapeutic outcomes, as shown by significantly shortened PFS (hazard ratio (HR) 11.29, p=0.002) and OS (HR 6.5, p=0.003) times in the high T2-EOR group as compared with the low T2-EOR group (**Figure 2**). This finding indicates that T2-EOR is negatively correlated with patient survival time and reflects a possibly divergent effect of SpTR on the outcomes of GBM patients receiving distinct therapeutic modalities.

### T2-EOR Was Negatively Correlated With TSIR Levels

**Figure 3:**
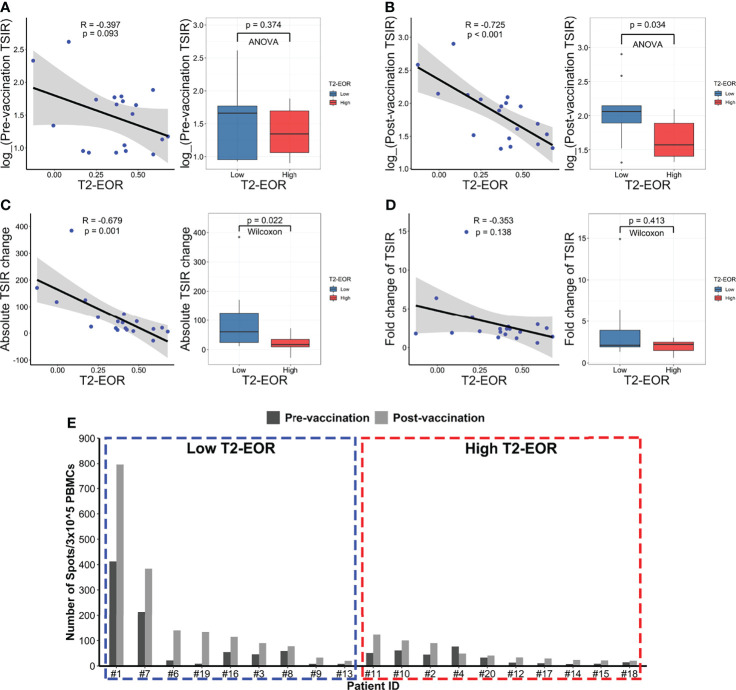
T2-EOR was negatively correlated with the levels of the tumour-specific immune response. Pearson correlation of T2-EOR with **(A)** pre-vaccination TSIR, **(B)** post-vaccination TSIR, **(C)** absolute change in TSIR from pre- to post-vaccination and **(D)** fold change in TSIR from pre- to post-vaccination. The differences in these TSIR indexes were also determined between the high and low T2-EOR groups using analysis of variance (ANOVA) or Wilcoxon nonparametric tests. **(E)** The levels of pre- and post-vaccination TSIR in each patient in this cohort are displayed as the high and low T2-EOR groups, respectively.

We next explored the mechanism underlying the link between T2-EOR and therapeutic outcomes. We hypothesized that T2-EOR would be negatively associated with TSIR, measured by an interferon-γ release ELISPOT assay on PBMCs, which has been shown to be closely associated with survival time (19). To test this hypothesis, we correlated T2-EOR with the levels of four indexes regarding TSIR: TSIR pre-vaccination (**Figure 3A**), TSIR post-vaccination (**Figure 3B**), absolute change in TSIR from pre- to post-vaccination (**Figure 3C**) and fold change in TSIR from pre- to post-vaccination (**Figure 3D**). We observed that the levels of post-vaccination TSIR (R=-0.725, p<0.001) and absolute TSIR change (R=-0.679, p=0.001) were negatively correlated with T2-EOR, whereas correlations between T2-EOR and pre-vaccination TSIR or fold change of TSIR did not reach a significant level. Accordingly, the high T2-EOR group exhibited reduced levels of post-vaccination TSIR and absolute TSIR change compared with the low group (**Figures 3B, C**). **Figure 3E** shows a distinct difference in post-vaccination TSIR levels between the two groups. Our previous study revealed high post-vaccination TSIR as a favourable prognosticator for patients’ PFS and OS (**Supplementary Figures 1A, B**) (30), and we also observed a similar trend in survival analysis for absolute TSIR change (**Supplementary Figures 1C, D**). Therefore, these findings indicate that the link between high T2-EOR and poorer outcomes could be attributed to its inverse correlation with the TSIR levels.

### High T2-EOR Was an Independent Predictor Associated With Shortened PFS and OS Times

**Figure 4:**
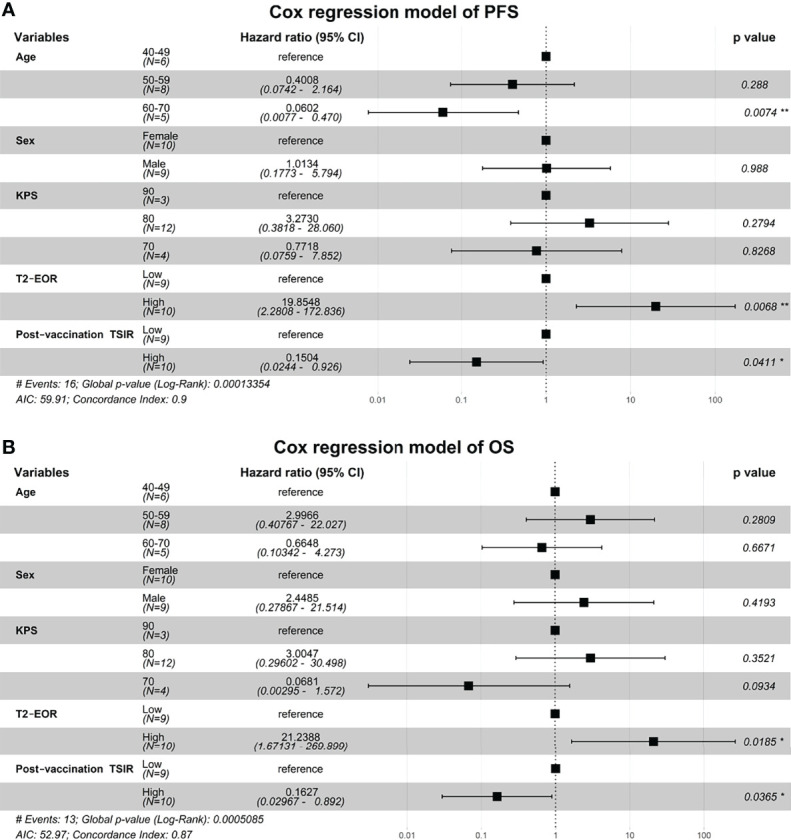
High T2-EOR was an independent predictor associated with shortened PFS and OS times. Two Cox regression models were fitted to identify independent prognosticators for **(A)** progression-free survival and **(B)** overall survival. *, P < 0.05. **, P < 0.01.

We fitted two Cox regression models, including age, sex, KPS score, T2-EOR and post-vaccination TSIR, to predict PFS and OS in this vaccinated cohort. As a result, both T2-EOR and post-vaccination TSIR emerged as significant independent prognosticators for PFS and OS (**Figures 4A, B**). High T2-EOR was significantly associated with shortened PFS (HR 19.85, p=0.0086) and OS (HR 21.24, p=0.0185) times. Compared with post-vaccination TSIR, T2-EOR can be measured prior to vaccination. Thus, T2-EOR is a potential biomarker that not only predicts immunotherapeutic outcomes but also facilitates the early recognition of patients who are more likely to benefit from the immunotherapy of HSPPC-96 vaccination.

## Discussion

Surgical resection remains a standard-of-care treatment for GBMs (32). Gross total resection of contrast-enhancing areas in GBMs has been widely reported to significantly extend PFS and OS in GBM patients receiving radiotherapy and temozolomide-based chemotherapy (33–36). Given the infiltrative growth nature of GBM, peritumoural oedema, shown as T2-nonenhancing region, also contains a small number of tumour cells, thus making it a region susceptible to tumour recurrence (11, 12). Therefore, SpTR, which leads to increased T2-EOR by minimizing T2-nonenhancing region, is theoretically associated with improved PFS and OS compared with gross total resection of contrast-enhancing areas. Recently, mounting evidence has supported the benefit of SpTR for the outcomes of glioma patients, with increased T2-EOR resulting in longer PFS and OS times (5, 9, 13, 35). However, the impact of T2-EOR on immunotherapeutic efficacy has not been elucidated. In this study, we reviewed the preoperative and postoperative MRI images of GBM patients who received HSPPC-96 vaccination in our previous clinical trial (NCT02122822). We revealed an inverse correlation of T2-EOR with vaccination-related antitumour immune response, represented as post-vaccination TSIR (**Figure 3B**) and absolute TSIR change (**Figure 3C**). Meanwhile, high T2-EOR was independently correlated with poorer clinical outcomes (**Figures 2**, **4**). Taken together, our results indicate that increased T2-EOR or SpTR has a detrimental impact on the immunotherapeutic efficacy of HSPPC-96 vaccination.

We proposed two possible mechanisms to explain the link of SpTR to worse immunotherapeutic efficacy. The first is that SpTR can reduce the residual tumour burdens, which are required to generate an effective immune response in immunotherapies such as CAR-T therapies (37), HSPPC-96 vaccination (27) and rindopepimut (a peptide vaccine against EGFR-vIII-expressing GBMs). Patients with a higher tumour burden at the time of CAR-T cell infusion exhibited better CAR-T cell persistence and proliferation (37). The HSPPC-96 vaccination trial on melanoma (27) revealed that the immunotherapy provided a significant benefit in patients with residual disease. The rindopepimut trial showed a possible long-term survival benefit with rindopepimut for GBM patients with significant residual disease (≥2 cm^2^ of residual enhancing tumour on post-chemoradiation imaging) compared with those with minimal residual disease (< 2 cm^2^ of residual enhancing tumour) (38). They suggested that residual disease is associated with greater antigen expression, which would lead to the enhanced anti-tumour immunity required for a therapeutic effect (38). In this study, we also observed a direct positive correlation between the T2-nonenhancing region residual volumes and TSIR levels (**Supplementary Figures 2A, B**), although this correlation did not have a significant impact on outcomes (**Supplementary Figures 2C, D**), likely due to the limited sample size of the study. Therefore, a larger sample-size trial with a prospective design is needed to further confirm this possible mechanism.

Another possible mechanism is that T2-EOR also represents a change in T2-nonenhancing region before and after surgery and thus could be an indicator reflecting the intensity of the immune response to surgery that would predict immunotherapeutic outcomes. In this scenario, T2-nonenhancing region, representing peritumoural oedema, is positively correlated with the immune response to surgical stress (31, 39). An increased T2-EOR reflects less change in postoperative T2-nonenhancing region than preoperative T2-nonenhancing region, indicating a less intensive immune response to surgeries and thus reflecting a lower native capacity to trigger the antitumour immunity required for immunotherapeutic effectiveness. However, to our knowledge, there is no direct evidence supporting this hypothesis, thus requiring further animal studies as well as early clinical trials to test it.

Again, given the limited sample size and retrospective design of this study, the current findings should be further confirmed in another larger and carefully designed cohort, although we observed a statistically significant association of T2-EOR with immunotherapeutic efficacy.

## Conclusion

In conclusion, to our knowledge, our study first revealed a significant association of SpTR or increased T2-EOR with worse immunotherapeutic outcomes in GBM patients, suggesting the potential of T2-EOR as an early biomarker for predicting the immunotherapeutic efficacy of HSPPC-96 vaccination.

## Data Availability Statement

The raw data supporting the conclusions of this article will be made available by the authors, without undue reservation.

## Author Contributions

Conceptualization, YZ and NJ. Methodology, XC. Software, XC. Formal analysis YZ and XC. Resources, NJ. Data curation, XC and YW. Writing—original draft preparation, XC. Writing—review and editing, YZ and NJ. Visualization, XC and CL. Supervision, YZ and NJ. Project administration, YZ and NJ. Funding acquisition, XH, HG, YZ and NJ. All authors have read and agreed to the published version of the manuscript.

## Funding

This research was funded by National Natural Science Foundation of China (81702451, 81930048), Capital characteristic Clinical Application Project (Z181100001718196), Capital Health Research and Development of Special Grant (2022-2-2047).

## Conflict of Interest

XH is a co-founder and shareholder of Cure & Sure Biotech Co., LTD, Shenzhen, China, 100070; HG is a shareholder of Cure & Sure Biotech Co., LTD, Shenzhen, China, 100070; Cure & Sure Biotech Co., LTD, Shenzhen, China, 100070 developed the HSPCC-96 vaccine and provided financial support to the vaccine trial (NCT02122822), on which the current study is based.

The remaining authors declare that the research was conducted in the absence of any commercial or financial relationships that could be construed as a potential conflict of interest.

## Publisher’s Note

All claims expressed in this article are solely those of the authors and do not necessarily represent those of their affiliated organizations, or those of the publisher, the editors and the reviewers. Any product that may be evaluated in this article, or claim that may be made by its manufacturer, is not guaranteed or endorsed by the publisher.
