# Supplementary material for: Resection of Noncontrast-Enhancing Regions Deteriorated the Immunotherapeutic Efficacy of HSPPC-96 Vaccination in Treating Glioblastoma
Source: Front Oncol. 2022 May 18;12:877190. doi: 10.3389/fonc.2022.877190 (PMC9158124; doi:10.3389/fonc.2022.877190)
Supplement: Supplementary file 1 [file DataSheet_1.docx]

Supplementary Material

# Supplementary Figures and Tables

Supplemental Table 1 displays the baseline characteristics of the included patients. Supplemental Table 2 shows the differences in the baseline characteristics between the high and low T2-EOR groups. Supplemental Fig. 1 shows the impacts of post-vaccination TSIR and absolute change of TSIR on clinical outcomes. Supplemental Fig. 2 shows the differences in post-vaccination TSIR and absolute change of TSIR between the high and low T2-RV groups and the impact of T2 residual volume on clinical outcomes.

## Supplementary Tables

Supplement Table 1. Baseline Characteristics

| Variables | Number of Patients | Proportion (%) |
| --- | --- | --- |
| Sex |  |  |
| Male | 9 | 47.4 |
| Female | 10 | 52.6 |
| Age at diagnosis, years |  |  |
| 40-49 | 6 | 31.6 |
| 50-59 | 8 | 42.1 |
| 60-70 | 5 | 26.3 |
| KPS |  |  |
| 90 | 3 | 15.8 |
| 80 | 12 | 63.2 |
| 70 | 4 | 21.1 |
| Median preoperative T2-NER volume, cm3 (range) | 75.69  (40.57-153.25) |  |
| Median postoperative T2-NER volume, cm3 (range) | 45.11  (24.60-154.15) |  |
| Median T2-EOR, % (range) | 40.1 (-12.7–67.8) |  |
| MGMT promoter status |  |  |
| Methylated | 2 | 10.5 |
| Nonmethylated | 14 | 73.7 |
| Unknown | 3 | 15.8 |
| IDH 1/2 mutations |  |  |
| Mutated | 2 | 10.5 |
| Wild-type | 14 | 73.7 |
| Unknown | 3 | 15.8 |
| TERT mutations |  |  |
| Mutated | 11 | 57.9 |
| Wild-type | 5 | 26.3 |
| Unknown | 3 | 15.8 |
| Status at last follow-up |  |  |
| Alive | 6 | 31.6 |
| Dead | 13 | 68.4 |

Supplement Table 2. Differences in Clinical Characteristics between the High and Low MRI T2-EOR Groups

| Variables | Low T2-EOR group (EOR < 40.1%) (N = 9) | High T2-EOR group (EOR ≥ 40.1%) (N = 10) | p value |
| --- | --- | --- | --- |
| Sex |  |  |  |
| Male | 2 | 7 | 0.06978 |
| Female | 7 | 3 |  |
| Age at diagnosis, years |  |  |  |
| 40-49 | 2 | 4 | 0.7272 |
| 50-59 | 4 | 4 |  |
| 60-70 | 3 | 2 |  |
| KPS |  |  |  |
| 90 | 0 | 3 | 0.2755 |
| 80 | 7 | 5 |  |
| 70 | 2 | 2 |  |
| MGMT promoter status |  |  |  |
| Methylated | 1 | 1 | 0.7771 |
| Nonmethylated | 6 | 8 |  |
| Unknown | 2 | 1 |  |
| IDH 1/2 mutations |  |  |  |
| Mutated | 1 | 1 | 0.7771 |
| Wild-type | 6 | 8 |  |
| Unknown | 2 | 1 |  |
| TERT mutations |  |  |  |
| Mutated | 4 | 7 | 0.5499 |
| Wild-type | 3 | 2 |  |
| Unknown | 2 | 1 |  |

## Supplementary Figures

**
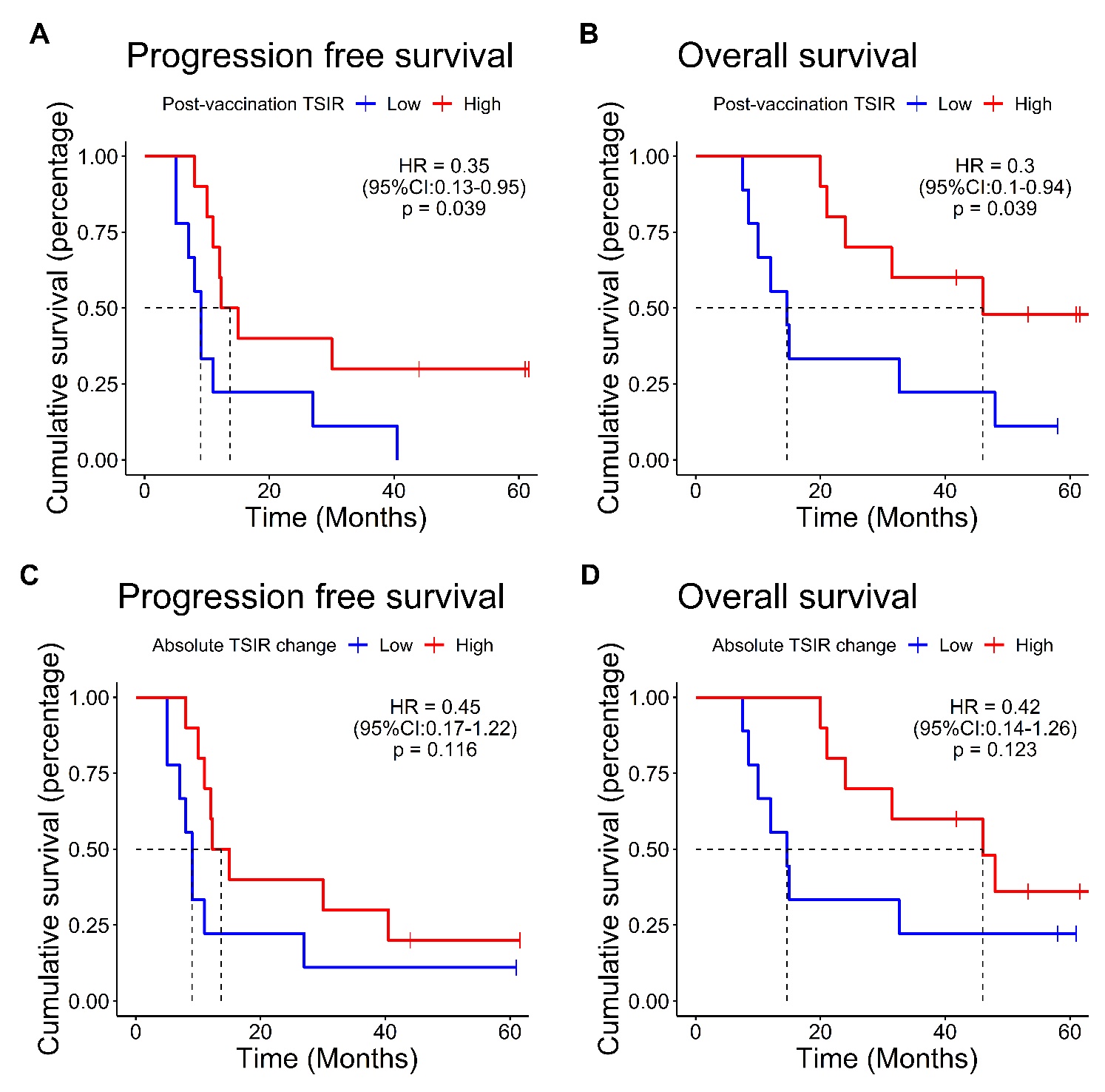
**

**Supplementary Figure 1.** Associations between TSIR indexes and prognosis. Kaplan–Meier estimates of (**A**, **C**) progression-free survival and (**B**, **D**) overall survival in 19 GBM patients divided into high and low post-vaccination TSIR/absolute change of TSIR groups. The log-rank test was applied to estimate the difference. Vertical lines indicate time points at which patients were censored.


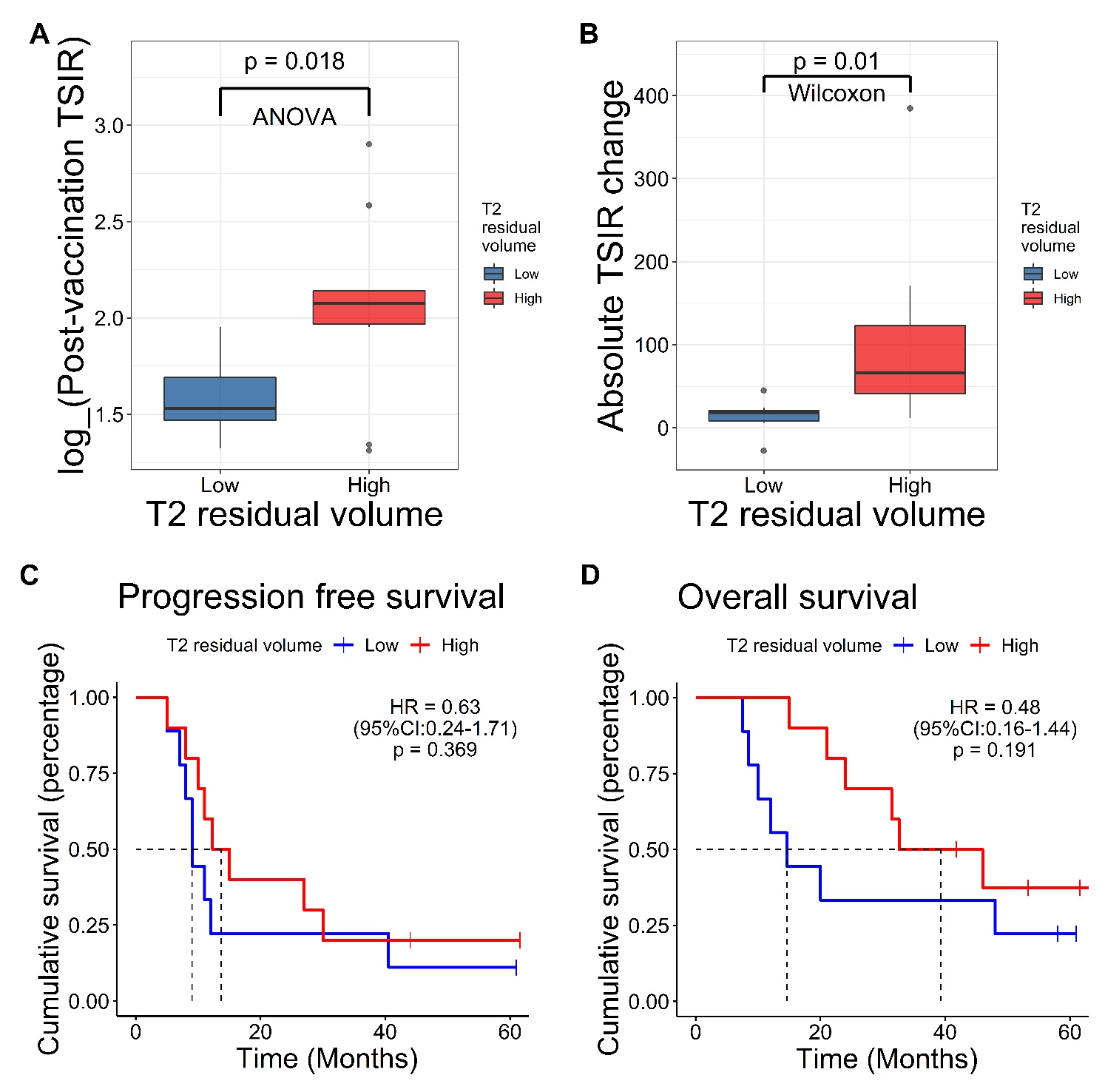


**Supplementary Figure 2.** Association between residual T2-NER volumes and TSIR indexes/survival. (**A** and **B**) Analysis of variance (ANOVA)/Wilcoxon nonparametric tests were performed between T2 residual volume and (**A**) post-vaccination TSIR and (**B**) absolute change in TSIR. (**C** and **D**) Kaplan–Meier estimates of (**C**) progression-free survival and (**D**) overall survival in 19 GBM patients divided into high and low T2 residual volume groups. The log-rank test was applied to estimate the difference. Vertical lines indicate time points at which patients were censored.
